# Alveolar socket surface area as a local risk factor for MRONJ development in oncologic patients on polypharmacy

**DOI:** 10.1007/s00784-025-06200-z

**Published:** 2025-02-08

**Authors:** Rellyca Sola Gracea, Isti Rahayu Suryani, Rocharles Cavalcante Fontenele, Hugo Gaêta-Araujo, Sonya Radi, Bahaaeldeen M. Elgarba, Sohaib Shujaat, Ruxandra Coropciuc, Reinhilde Jacobs

**Affiliations:** 1 OMFS-IMPATH Research Group, Department of Imaging & Pathology, Faculty of Medicine, KU Leuven & Department of Oral and Maxillofacial Surgery, University Hospitals Leuven, Leuven, Belgium; 2 Department of Dentomaxillofacial Radiology, Faculty of Dentistry, Universitas Gadjah Mada, Yogyakarta, Indonesia; 3 Department of Stomatology, Public Health and Forensic Dentistry, Division of Oral Radiology, School of Dentistry of Ribeirão Preto, University of São Paulo, São Paulo, Ribeirão Preto Brazil; 4 Department of Prosthodontics, Faculty of Dentistry, Tanta University, Tanta, Egypt; 5 King Abdullah International Medical Research Center, Department of Maxillofacial Surgery and Diagnostic Sciences, College of Dentistry, King Saud bin Abdulaziz University for Health Sciences, Ministry of National Guard Health Affairs, Riyadh, Kingdom of Saudi Arabia; 6 Department of Dental Medicine, Karolinska Institutet, Alfred Nobels Allé 8, Huddinge, Stockholm, 141 50 Sweden; 7 OMFS-Impath Research Group, KU Leuven, Kapucijnenvoer 7, Leuven, B-3000 Belgium

**Keywords:** Tooth extraction, Polypharmacy, Tooth socket, Wound healing, Jaw, Osteonecrosis

## Abstract

**Objectives:**

To determine the impact of alveolar socket surface area and number of root extractions for developing medication-related osteonecrosis of the jaw (MRONJ) in polypharmacy patients following multiple tooth extractions.

**Materials and methods:**

A retrospective sample of 40 patients was recruited, including 20 polypharmacy patients (109 tooth extractions) who developed MRONJ in at least one of the extraction sites, matched with 20 controls (100 tooth extractions). Tooth-specific alveolar socket surface areas were assessed using CBCT scans from the control group to establish reference values for alveolar socket surface areas in polypharmacy patients with MRONJ. Correlations between the number of extracted tooth roots, alveolar socket surface area, and development of MRONJ were analysed within the polypharmacy group.

**Results:**

40% of tooth extractions in polypharmacy patients undergoing multiple extractions resulted in the development of MRONJ, with a higher prevalence observed in the mandible (44%). Among the extracted mandibular tooth roots, half were susceptible to MRONJ, and 45% of the exposed socket surface area was affected. Both jaws showed an increased risk (20%) of MRONJ following molar extraction. A strong positive correlation existed between extraction sites that developed MRONJ and both the number of mandibular tooth roots extracted (r = + 0.861; *p* < 0.001) and the total exposed alveolar socket surface area (r = + 0.757; *p* < 0.001). However, no significant correlations were observed in the upper jaw.

**Conclusions:**

This study is the first to demonstrate that both mandibular alveolar socket surface area and number of extracted tooth roots are positively related to extraction sites developing MRONJ in polypharmacy patients undergoing multiple tooth extractions.

**Clinical relevance:**

Identifying high-risk patients and implementing preventive strategies can reduce MRONJ incidence, underscoring the need for careful management of polypharmacy patients, especially those undergoing mandibular tooth extractions.

**Supplementary Information:**

The online version contains supplementary material available at 10.1007/s00784-025-06200-z.

## Introduction

Tooth extraction is a common procedure where the healing outcome is crucial to overall health [1]. The alveolar socket, also referred to as a dental socket or alveolus, is a cavity within the jawbone that accommodates the tooth and is integral to the healing process [2]. The healing of the alveolar socket involves a series of internal and external processes aimed at wound closure and establishment of tissue homeostasis [3].

In recent years, there has been a surge in studies investigating the intricate factors influencing healing post-extraction, especially in patients with polypharmacy, i.e., the concurrent use of multiple medications [4, 5]. Indeed, the combination of medications can impact physiological processes, potentially affecting the body’s recovery ability from various medical procedures, such as dental extraction [6]. Patients on multiple medications, particularly bone-modifying agents, are at an elevated risk of delayed wound healing and developing medication-related osteonecrosis of the jaw (MRONJ) [7]. For instance, bisphosphonates are well-known agents with potent inhibitory effects on osteoclastic activity [8, 9]. In patients treated with these medications, tooth extraction can result in the alveolar bone’s inability to form new bone. The overlying bone, deprived of blood supply from the underlying bone is prone to deterioration, leading to clinically exposed bone [10]. In addition, non-antiresorptive agents such as chemotherapy, antiangiogenic tyrosine kinase inhibitors, and corticosteroids have also been associated with MRONJ development [11, 12].

MRONJ is characterised by the presence of exposed bone or bone that can be probed through an intraoral or extraoral fistula in the maxillofacial region, persisting for more than 8 weeks in patients with no prior history of radiotherapy to the head and neck region [13]. Extensive research has focused on identifying the clinical risk factors and early radiographic signs that may precede the onset of MRONJ. Clinical risk factors associated with MRONJ include medical comorbidities such as the stage of cancer, chemotherapy, antiresorptive drug use, targeted therapy, systemic inflammatory disease, and tobacco use. Dental comorbidities include tooth extraction, periodontal disease, dental implants, oral surgery, and trauma [14]. Additionally, radiographic signs such as bone sclerosis, osteolytic areas, lamina dura thickening, persistent alveolar socket, periapical radiolucency, thick mandibular cortex, wide periodontal ligament space, periodontal bone loss, and enlargement of the mandibular canal have also been identified as early indicators of MRONJ development [15].

Local risk factors for MRONJ development associated with the defect size following multiple tooth extractions have been rarely investigated [16]. Large defects resulting from multiple tooth extractions, reconstructive surgery of congenital defects, trauma, or tumour, as well as pre-prosthetic reconstructive surgery, are among the local factors influencing wound healing in the oral cavity [17]. Regarding tooth-specific types, it is known that molars require a longer healing time. Kim et al. reported that erratic socket healing was more commonly observed at molar sites compared to premolar sites, with a prevalence of 5% and 3%, respectively [18]. Furthermore, underlying bony conditions (e.g., endodontic and periodontal disease) in the area where teeth are extracted increase the likelihood of developing MRONJ [16]. From this perspective, it is vital to gain insight into the increased risk of MRONJ development based on the wound size resulting from multiple tooth extractions. This study hypothesized that patients with polypharmacy undergoing multiple tooth extractions are at a higher risk of developing MRONJ in multiple extraction sites.

The primary aim of this study was to evaluate how the development of MRONJ at extraction sites relates to the total alveolar socket surface area affected by multiple tooth extractions in patients undergoing polypharmacy. The secondary aim was to assess the impact of the number of extracted tooth roots on the development of MRONJ at these sites.

## Methods

This retrospective follow-up study was conducted in compliance with the World Medical Association Declaration of Helsinki on medical research. Ethical approval was obtained from the Ethical Review Board of the University Hospitals Leuven (reference number: S57824). Informed consent was deemed unnecessary, as patient-specific information was anonymised. A priori power analysis was performed using G*power software (G*Power, Version 3.1.9.2, Düsseldorf, Germany) to ascertain the appropriate sample size. This analysis was predicated on an anticipated effect size of 0.35, a significance level of α = 0.05, and a desired statistical power of 1 - β = 0.80. The results indicated that a minimum sample size of 84 teeth was necessary for the study.

**Fig. 1:**
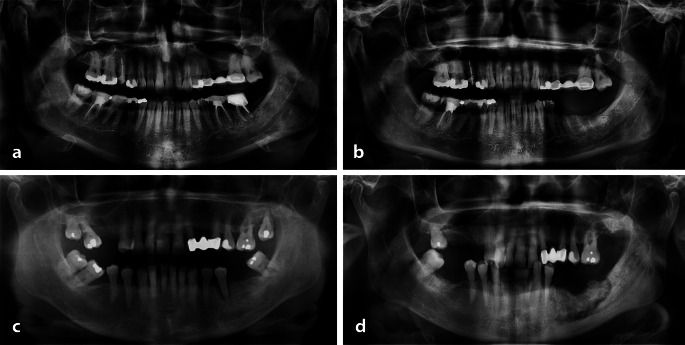
Panoramic radiograph of control and polypharmacy patients following multiple tooth extractions. Control patient: (**a**) pre-extraction image of teeth 36 and 37; (**b**) normal healing of alveolar socket at extraction sites of teeth 36 and 37. Polypharmacy patient: (**c**) pre-extraction of teeth 17, 28, 37, 35, 31, 41, 42, 47; (**d**) MRONJ observed at extraction sites of teeth 31, 41, 35, 37 and normal extraction site healing at 17, 28, 42, 47

Medical records and dental reports of patients who underwent tooth extraction at the Department of Oral and Maxillofacial Surgery, UZ Leuven, Belgium were examined. Inclusion and exclusion criteria are listed in Table 1. A total of 40 patients who underwent multiple tooth extractions were divided into two groups based on medication use: 20 patients with polypharmacy matched for age and tooth extraction with 20 healthy patients serving as controls. Multiple extraction was defined as the removal of at least two teeth in any quadrant of the upper and/or lower jaw, regardless of whether the teeth were adjacent. The polypharmacy group was composed of patients who were regularly taking five or more medications before the extraction. These included a combination of bone-modifying agents, such as antiresorptive drugs (e.g., bisphosphonates and denosumab) and non-antiresorptive drugs (e.g., chemotherapy, corticosteroids, hormone therapy, and immunosuppressive agents), as well as additional medications for other systematic health conditions. All 20 patients in the polypharmacy group developed MRONJ in at least one of the extraction sites. The control group consisted of healthy patients who did not receive any medications prior to extraction and demonstrated uneventful socket healing (i.e., no need for recall or reintervention). All patients in the control group underwent pre-extraction cone beam computed tomography (CBCT) scans, justified for other pre-surgical interventions (e.g., pre-surgical planning for implant placement). Figure 1 illustrates the pre- and post-extraction panoramic radiographs of patients belonging to control and polypharmacy groups.

Patients received prophylactic antibiotics starting 2–3 days prior to the extraction, and antimicrobial mouth rinses were also used for 7–10 days before the procedure. Extractions were performed under local anesthesia using elevators and forceps. Following extraction, meticulous curettage was conducted, and the socket was irrigated with normal saline. In half of the cases within the polypharmacy group, leukocyte-platelet rich fibrin (L-PRF) membranes were placed to promote healing and tissue regeneration. The extraction site was sutured using 3 − 0 vicryl resorbable sutures. Prophylactic antibiotics were continued until the first follow-up visit. The prescribed antibiotic regimen consisted of amoxicillin 875 mg/clavulanic acid 125 mg or clindamycin 300 mg three times per day for approximately 2 to 3 weeks post-extraction. After this initial treatment period, antibiotic therapy was adjusted to a maintenance dose of amoxicillin 500 mg or doxycycline 100 mg per day, continuing until the infection subsided or mucosal healing was achieved. Follow-up visits were initially scheduled every two weeks to assess healing progress, with subsequent visits arranged monthly or every three months depending on the patient’s condition.

**Table 1:** Inclusion and exclusion criteria for patients in either polypharmacy or control groups

Criteria	Polypharmacy	Control
**Inclusion**		
1. Polypharmacy: concurrent use of five or more medications, including at least one bone-modifying agents along with other medications.	✓	✗
2. Panoramic radiograph prior to extraction	✓	✓
3. Panoramic radiograph 6 months post-extraction	✓	✓
4. Multiple extractions: minimum two teeth	✓	✓
5. Clinical diagnosis of extraction socket healing as either normal healing or MRONJ development	✓	✓
6. CBCT available prior to extraction	✗	✓
**Exclusion**		
1. Patients with previous history of head and neck radiotherapy	✓	✓
2. No panoramic radiograph prior to tooth extraction	✓	✓
3. Panoramic radiograph with low image quality	✓	✓

MRONJ = medication-related osteonecrosis of the jaw

(✓) = applicable

(✗) = not applicable

Clinical characteristics of the patients were extracted from medical records and dental reports, including factors such as age, gender, and primary disease. The patients’ medication status was documented, classifying them as either on multiple medications (polypharmacy) or not on any medication. Details regarding the extracted teeth were recorded, including the number of teeth extracted, their location in the upper or lower jaw, and the type of tooth (incisor, canine, premolar, molar). Post-extraction outcomes were categorized into normal healing or MRONJ occurrence. Normal healing was assessed based on the evaluation of mucosal healing within an 8-week period following extraction. During this time, patients were closely monitored through follow-up visits scheduled every two weeks. The diagnosis of MRONJ was established through clinical observations and panoramic radiographic data (Vistapano, Dürr Dental, Bittingheim-Bissingen, Germany) obtained during the patients’ 6-month follow-up.

The present study aimed to establish whether the number of tooth roots extracted, and the related exposed alveolar socket surface area impacted osteonecrosis development in polypharmacy patients. While the number of tooth roots extracted could be assessed using two-dimensional (2D) image data, three-dimensional (3D) image data is a prerequisite for assessing the alveolar socket surface area. Considering that the present retrospective study was based on pre- and post-extraction panoramic radiographs, the available 2D image data could only aid in assessing the relation between the number of tooth roots extracted and the eventual outcome of the alveolar socket healing. However, for establishing a relation between extraction socket healing and alveolar socket surface, benchmark values were obtained from pre-extraction CBCT image data of the control group. This approach allowed obtaining a relative reference for alveolar socket area of the respective tooth extraction sites in both control and polypharmacy patients, given the fact that the presence of pre-extraction CBCT was not a criterion for patient inclusion to the polypharmacy group. The CBCT images were captured using the Newtom VGI evo device (Cefla, Imola, Italy) prior to extraction, saved in the Digital Imaging and Communication in Medicine (DICOM) format, and subsequently imported into an online cloud-based platform known as ‘Virtual Patient Creator’ (creator.relu.eu, version 3.12, Relu BV, Leuven, Belgium). This platform was used to generate segmentations of the jawbone and teeth, which allowed for the creation of virtual 3D models in Standard Tessellation Language (STL) format. These 3D models were then imported into the Mimics Innovation Suite (version 24.0, Materialise N.V., Leuven, Belgium) to calculate the surface area of the alveolar socket, following the methodology described by Regnstrand et al. [19]. Manual segmentation of the alveolar socket’s 3D digital models was performed at the crestal bone level, specifically 1.50 mm apical to the cementoenamel junction (CEJ) to the lowest point of the roots in the axial, coronal, and sagittal planes. Thereafter, the alveolar socket at the time of extraction was simulated, and the surface area of socket was automatically calculated in square millimetres (mm²). Average measurements were calculated from 10 sockets for each specific tooth type in both the upper and lower jaws. This resulted in a total of 80 measurements of alveolar socket surface area.

Observer reliability for surface area measurements was carried out on a subset comprising 10% of the total sample. Two maxillofacial radiologists (IRS and RSG), each with more than 5 years of experience, independently and blindly conducted the assessments twice. The observations were repeated at an interval of one week to compute both intra- and inter-observer reliability.

### Statistical analysis

Statistical analysis was performed using R (version 4.4.0, R Core Team, Vienna, Austria) in conjunction with RStudio (2023.12.1 + 402). The intra- and inter-observer reliability for the measurement of alveolar socket surface area in the control group was evaluated using the intraclass correlation coefficient (ICC). Descriptive statistics were reported for all the data. Pearson correlation analyses were performed within the polypharmacy group to examine the correlation between the total number of extracted tooth roots and the number of extraction sites that developed MRONJ. This statistical method was also employed to evaluate the relationship between the total exposed alveolar socket surface area following multiple tooth extractions and the alveolar socket surface area of the extraction sites that developed MRONJ within the same jaw for each patient. A p-value of less than 0.05 was considered statistically significant.

## Results

Patients who fulfilled the inclusion criteria were retrospectively identified from records spanning the period from 2007 to 2018. Table 2a provides an overview of patient characteristics and the details of the teeth extracted within the polypharmacy group. The ages of the patients varied from 47 to 85 years, with a majority being female. Out of the 109 teeth that were extracted, 44% from the lower jaw resulted in the development of MRONJ, primarily in the molar region. Zoledronic acid was the most commonly used anti-resorptive drug (*n* = 9), whereas hormone therapy was the most frequently administered non-anti-resorptive drugs (*n* = 15). Three patients received monoclonal antibodies, with the specific type described in Supplementary Table 1. Half of the patients received L-PRF treatment, which was applied to 54 extracted teeth. Supplementary Table 2 outlines the use of L-PRF and the MRONJ outcomes for each patient, showing no significant difference in the rate of MRONJ development between patients with and without L-PRF. Additionally, a descriptive analysis of the number of tooth roots extracted revealed that approximately 37% of the total roots extracted from the upper jaw and nearly 46% from the lower jaw were susceptible to MRONJ. Table 2b delineates the characteristics of the control group. The ages of the patients ranged from 46 to 89 years, with a female-to-male ratio of 1:2. A total of 100 extracted teeth were included, that were almost equally distributed across different types of teeth. All teeth extracted in the control group demonstrated normal healing.

**Table 2a:** Characteristics of patients and extracted teeth in the polypharmacy group

**Characteristics of polypharmacy patients**							
Number of patients, n				20			
Age, years (mean ± SD)				67 ± 10.8			
Gender, n		Male		9			
		Female		11			
Primary cancer, n		Breast cancer		9			
		Prostate cancer		6			
		Multiple myeloma		3			
		Lung cancer		2			
Drugs used, n		ARD	Zoledronic acid	11			
			Ibandronat	1			
			Pamidronate	1			
			Denosumab	7			
		Non-ARD	Chemotherapy agent	9			
			Monoclonal antibody	3			
			Corticosteroid	13			
			Hormone therapy	15			
Time on ARD, months (mean ± SD)				21 ± 18			
Use of L-PRF		Yes		10 (54 teeth)			
		No		10 (55 teeth)			
**Characteristics of extracted teeth on MRONJ**							
Number of extracted teeth, n (%)				109 (100%)			
MRONJ development, n (%)				n	MRONJ+	MRONJ–	
				109	43 (40)	66 (60)	
Jaw position, n (%)		Upper jaw		54	19 (35)	35 (65)	
		Lower jaw		55	24 (44)	31 (56)	
Number root extracted, n (%)		Upper jaw		117	43 (37)	74 (63)	
		Lower jaw		76	35 (46)	41 (54)	
Tooth type, n	Upper	Incisor		54	1	5	
		Canine			3	4	
		Premolar			4	7	
		Molar			11	19	
	Lower	Incisor		55	7	6	
		Canine			2	4	
		Premolar			4	11	
		Molar			11	10	
Periodontal bone loss, n	Upper	Incisor		43	1	3	
		Canine			3	2	
		Premolar			4	4	
		Molar			10	16	
	Lower	Incisor		37	7	4	
		Canine			0	2	
		Premolar			4	6	
		Molar			8	6	

ARD = anti-resorptive drugs

BPs = bisphosphonates

SD = standard deviation

(+) = MRONJ development observed

(–) = No MRONJ development observed

**Table 2b:** characteristics of patients and extracted teeth in the control group

**Characteristics of control patients**				
Number of patients, n			20	
Age, years (mean ± SD)			62 ± 11.4	
Gender, n	Male		14	
	Female		6	
**Characteristics of extracted teeth**				
Number of extracted teeth, n (%)				100 (100%)
Tooth type, n (%)	Upper	Incisor	13 (26)	
		Canine	11 (22)	
		Premolar	10 (20)	
		Molar	16 (32)	
	Lower	Incisor	14 (28)	
		Canine	7 (14)	
		Premolar	14 (28)	
		Molar	15 (30)	

**Table 3:**
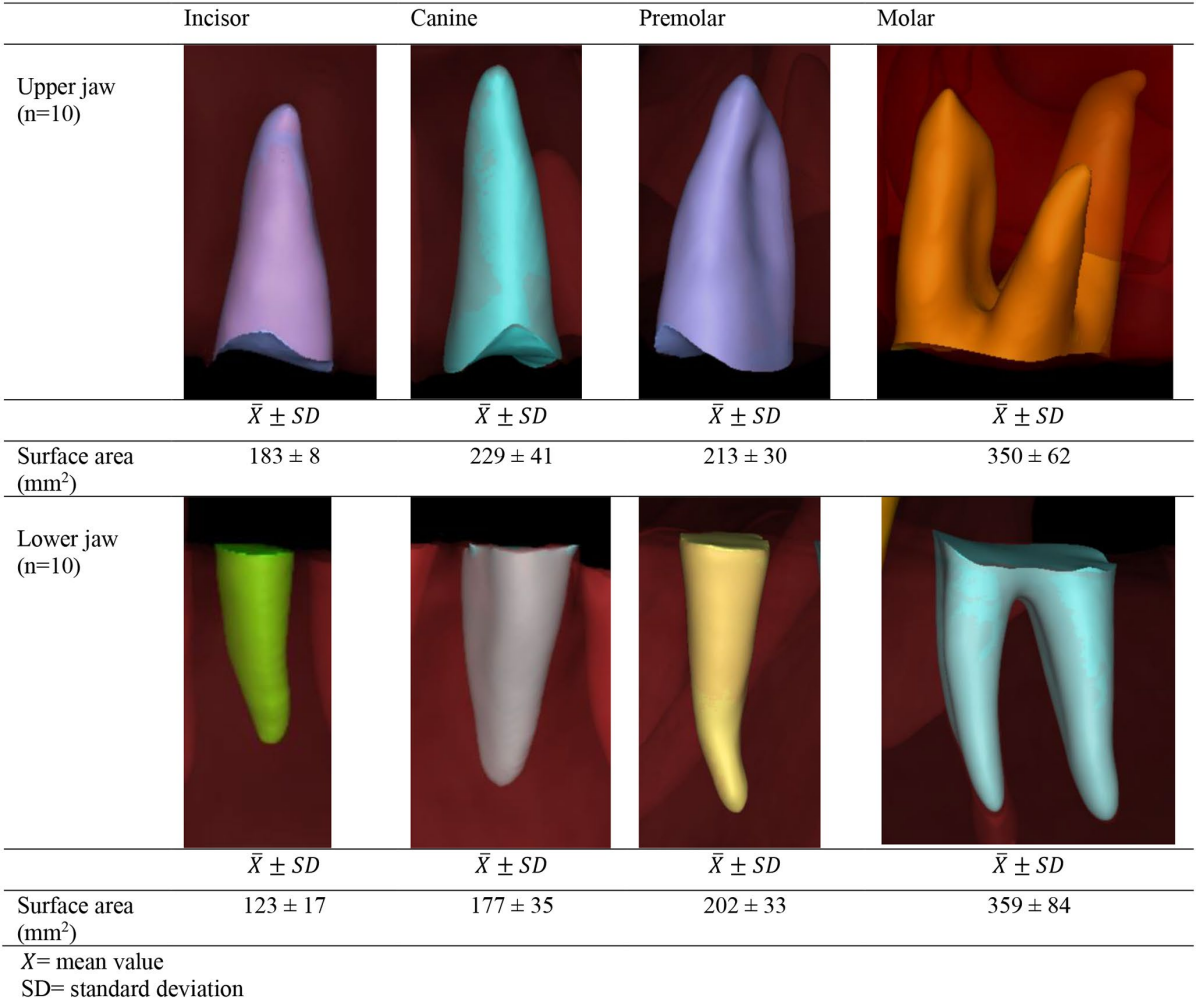
3D simulation of tooth-specific alveolar socket and related average alveolar socket surface area

Table 3 with pictorial models lists the measurements of the alveolar socket surface area for upper and lower incisors, canines, premolars, and molars. Both intra-rater and inter-rater reliability for these measurements were found to be high, with values of 0.9 (95% CI 0.9-1) and 0.9 (95% CI 0.8–0.9), respectively. Molar teeth had the largest average alveolar socket surface area. Specifically, the extraction of upper molars resulted in an exposed alveolar socket surface area averaging 350 ± 62 mm², while the extraction of lower molars averaged 359 ± 84 mm².

**Fig. 2:**
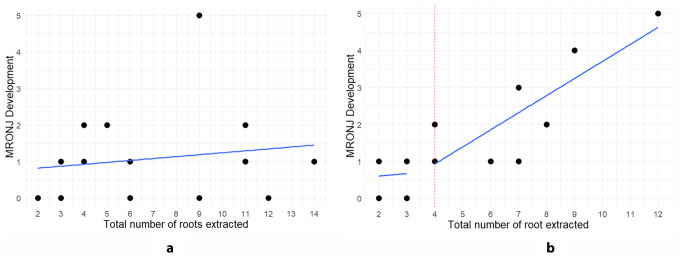
Scatter plots depicting distribution of extraction site with MRONJ development and total number of tooth roots extracted, in upper jaw (**a**) and lower jaw with cutoff value for increased risk of MRONJ development (**b**)

Figure 2 displays scatter plots showing the distribution of extraction sites that developed MRONJ, and total number of tooth roots extracted within the polypharmacy group, categorized by upper and lower jaw. Pearson correlation analysis showed no significant correlation in the upper jaw, while a strong positive correlation (r = + 0.861, *p* < 0.001) was observed in the lower jaw. Notably, a cutoff value of 4 roots was identified, which suggests that the extraction of 4 or more tooth roots significantly increased the risk of MRONJ development.

**Fig. 3:**
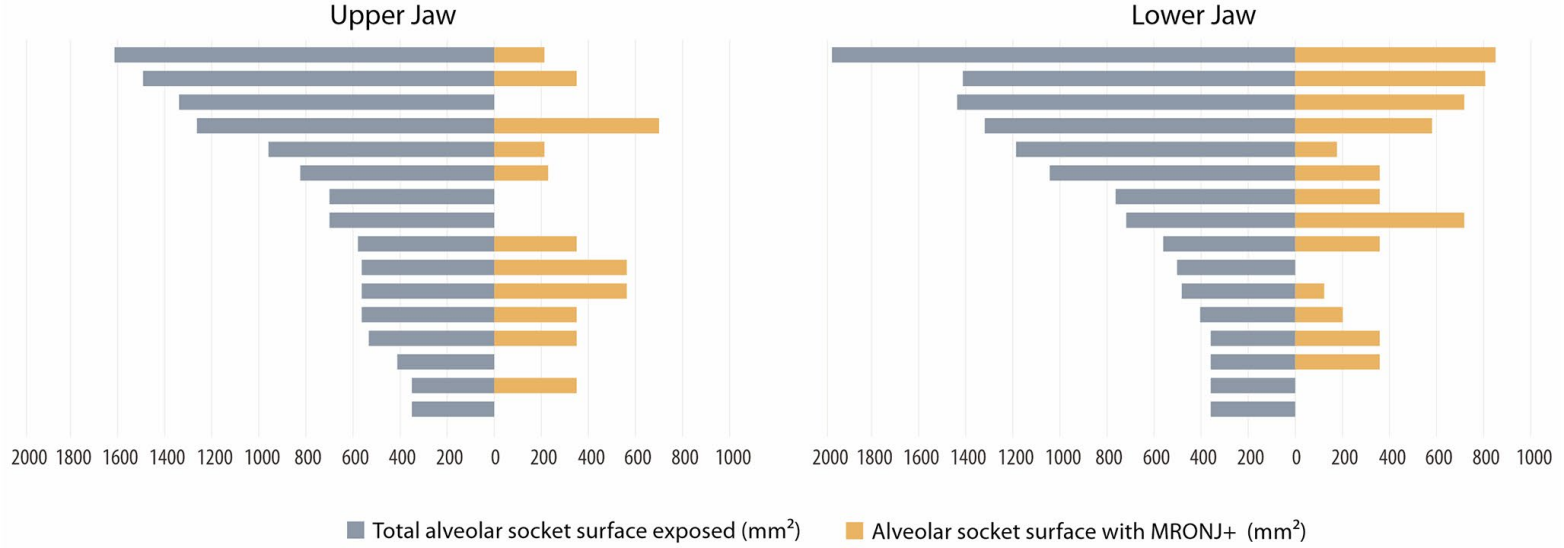
Horizontal bar chart shows surface area of teeth with MRONJ development versus total alveolar socket surface area exposed after multiple tooth extractions in either upper (**a**) or lower jaw (**b**)

The evaluation of the alveolar socket surface area following multiple tooth extractions in the polypharmacy patients showed that MRONJ development occurred in 36% of the exposed alveolar surface area in the upper jaw and 45% in the lower jaw. Figure 3 illustrates the distribution of the alveolar socket surface area in the polypharmacy group. Pearson correlation analysis showed no significant correlation for multiple extractions in the upper jaw of polypharmacy patients. A strong positive correlation (r = + 0.757, *p* < 0.001) was observed between the total exposed alveolar socket area and the alveolar socket area developing MRONJ after multiple tooth extractions in the lower jaw of these patients.

## Discussion

The healing process of extraction sockets is influenced by a variety of factors, including local and systemic conditions, iatrogenic influences, and environmental elements [20, 21]. The primary objective of this study was to evaluate the effect of multiple tooth extractions on the development of MRONJ in patients on multiple medications. This retrospective study was designed to focus on local factors contributing to the development of MRONJ in polypharmacy patients, particularly alveolar socket surface area and the number of tooth extractions. A within-patient analysis was selected because many patients had multiple extraction sites with varying outcomes—some sites developed MRONJ, while others healed normally. This approach minimized the potential bias from patient-specific risk factors, such as medication type, duration of treatment, or underlying health conditions [16].

The present study is the first to analyse the 3D alveolar socket surface area and its relationship to the development of MRONJ in polypharmacy patients. CBCT scans from healthy patients were used as a benchmark to assess the alveolar socket surface area. Although a direct comparison between polypharmacy and non-polypharmacy patients who are at risk of MRONJ might enhance the robustness of the findings, the procurement of pre-extraction CBCT scans from those multiple medications poses significant challenges. These challenges arise because such patients rarely undergo 3D radiological assessments before dental extractions, thereby hindering the establishment of an ideal reference dataset. Although the methodology applied was innovative, it should be noted that Agbaje et al. had previously described volumetric CBCT analysis as a clinically valuable tool for assessing delayed or impaired wound healing of extraction sockets in patients who had undergone radiation therapy [22, 23].

In the present study, both upper and lower molars were found to have a larger surface area and a higher risk of MRONJ development compared to other teeth. The average alveolar socket size of the molars aligned with the findings of Lakhani et al., who reported an average surface area of 391 ± 435 mm^2^ and 375 ± 331 mm^2^ for lower and upper first molar, respectively [24]. Although no significant correlation existed in the upper jaw, a significant positive correlation was observed between the total number of tooth roots extracted in the lower jaw and the extraction sites that developed MRONJ. Half of the extracted mandibular tooth roots developed MRONJ in polypharmacy patients who underwent multiple tooth extractions. This observation was further supported by a significant positive correlation between the alveolar socket surface area and MRONJ, as well as the total surface area exposed following multiple mandibular tooth extractions in patients on multiple medications. As the number of extracted tooth roots increased, so did the exposed surface area of the alveolar socket, demanding greater healing capacity from the body. This increased demand for healing must be considered in the context of decreased vascularization and salivary flow, particularly in patients on multiple medications with chronic dental infections necessitating extraction [12, 16, 17, 25].

The analysis identified a cutoff value of four extracted tooth roots, indicating that extracting four or more tooth roots increases the risk of MRONJ development in the lower jaw. This suggests that a larger extraction wound or alveolar socket increases the risk of developing MRONJ. This finding was consistent with the observations of Buchbender et al., who reported that the risk profile of MRONJ was highest for osteotomy interventions (14%), followed by multiple extraction (11%) and single extraction (5%) [26]. However, it should be noted that the authors did not exclusively focus on patients taking multiple medications.

Numerous studies have identified the mandible as being more prone to the development of MORNJ [27–34]. Notably, the mandible, especially in the posterior region, possesses unique characteristics compared to long bones, thereby creating a distinct environment. In contrast to long bones, which are primarily formed by endochondral ossification, the development of the mandible bone is largely attributed to intramembranous ossification. Additionally, the mandibular bone contains a higher proportion of collagen. These specific anatomical characteristics make the mandibular jawbone more susceptible to osteonecrosis [35]. The cortication of the mandible may be more susceptible to the effects of multiple medications, impacting the osteoclastic activity. This susceptibility, in an environment with altered salivary flow mechanisms and a decreased immune response, heightens the likelihood of developing MRONJ [25, 35]. Moreover, the patterns of vascularization, which significantly vary between the maxilla and mandible, play a crucial role in the healing of alveolar sockets and the development of MRONJ. The maxilla exhibits more extensive vascularization, with blood vessels distributed throughout the bone. This enhanced vascularization facilitates a faster and more efficient recovery process [36]. In contrast, the mandible has limited vascularization, with blood vessels primarily running through canals. Consequently, mandibular extractions are more challenging and result in slower healing [37]. Vascularization is vital as it supplies bone cells with essential elements such as oxygen, nutrients, hormones, and growth factors, all of which are critical for bone regeneration and remodelling. It also plays a role in the transportation of medications, including antibiotics. Impaired vascularization can result in abnormal healing, which may ultimately lead to osteonecrosis [38].

The occurrence of MRONJ in polypharmacy patients is complex and influenced by several interconnected factors. One such factor is the disruption of local immune responses. Numerous studies have underscored the importance of the mucosal immune system in protecting against microbial threats and maintaining balance, especially after dental injuries or in the presence of chronic periodontitis [39, 40]. The process of bone invasion during multiple tooth extractions compromises the integrity of the mucosal epithelial barrier, thereby enabling increased bacterial invasion and colonisation. This heightened bacterial activity could potentially lead to jawbone infection, particularly in instances of mucosal ulceration and periodontal disease, which are acknowledged as the initial pathological events of MRONJ [41]. Furthermore, in patients treated with zoledronate, the local immune response within the oral cavity may be disrupted due to the inhibition of dendritic cell differentiation and function. This disruption creates a more conducive environment for bacterial colonisation, thereby escalating the risk of subsequent MRONJ development [42]. In the context of the osteoimmune response, bone-modifying agents affect the interaction between the immune system and bone, leading to an imbalance in bone homeostasis and low bone turnover. This low bone turnover allows for the accumulation of microdamage in the jaw and opportunities for bacterial colonisation, a mechanism proposed as a contributing factor to the development of MRONJ [43]. Another crucial factor to consider is genetic predisposition. It has been suggested that genetic variations among individuals may either amplify or mitigate the risk of MRONJ [44]. Specifically, Matrix Metalloproteinase-2 (MMP-2), a protein predominantly found in human tissue, has been implicated as a potential gene increasing susceptibility to MRONJ induced by bisphosphonates [45].

While the existing data offers valuable insights into the increased risk of MRONJ following multiple tooth extractions in patients on polypharmacy, several limitations warrant consideration. The retrospective design of the study, coupled with a limited sample size and reliance on single-centre data, may introduce sampling bias and restrict the generalizability of the findings. Moreover, using CBCT data from healthy individuals as a benchmark, rather than from patients at risk for MRONJ, presents a significant limitation. Ideally, having access to pre-extraction CBCT scans from patients at risk for MRONJ would improve the accuracy of risk assessments and provide more robust data to inform clinical decision-making. Future research should aim to overcome these limitations by conducting larger, prospective studies that include comprehensive imaging data. This should encompass pre-extraction CBCT scans from patients predisposed to MRONJ, thereby enhancing the understanding and management of this condition. Incorporating salivary and genetic testing, along with close monitoring of both mucosal wound healing and underlying bone remodelling, could provide a more thorough understanding of MRONJ development in polypharmacy patients Furthermore, although L-PRF is a promising treatment approach that facilitates tissue healing and bone regeneration [46], the present study could not directly compare extraction sites with and without L-PRF application. This limitation is due to the retrospective nature of the study and the fact that all patients in the polypharmacy group developed MRONJ, further limiting the ability to evaluate the protective role of L-PRF. Further randomised trials may help to verify the present observations and to develop patient-specific risk assessment and management strategies for the healing of tooth extractions in clinical practice.

In conclusion, the current study provides preliminary evidence that multiple tooth extractions in polypharmacy patients may increase the risk of MRONJ development. It is the first to establish that both mandibular alveolar socket surface area and number of extracted tooth roots show a positive relationship with MRONJ development in polypharmacy patients. Importantly, among polypharmacy patients who underwent multiple mandibular tooth extractions, up to half of the extracted roots developed MRONJ.

## Electronic supplementary material

Below is the link to the electronic supplementary material.

Supplementary Material 1

## Data Availability

Data supporting the results of this study can be obtained from the corresponding author upon reasonable request.
