# Supplementary material for: Alveolar socket surface area as a local risk factor for MRONJ development in oncologic patients on polypharmacy
Source: Clin Oral Investig. 2025 Feb 8;29(2):123. doi: 10.1007/s00784-025-06200-z (PMC11805769; doi:10.1007/s00784-025-06200-z)
Supplement: Supplementary file 1 — Supplementary Material 1 [file 784_2025_6200_MOESM1_ESM.docx]

Supplementary Table 1. ARD and non-ARD medications used by each patient.

| Patient ID | ARD | | | | Non-ARD | | | |
| --- | --- | --- | --- | --- | --- | --- | --- | --- |
|  | Zoledronic acid | Denosumab | Ibandronat | Pamidronat | Chemo-therapy | Monoclonal antibody | Cortico-steroid | Hormon therapy |
| 1 | ✓ |  |  |  | ✓ |  |  |  |
| 2 |  | ✓ |  |  |  |  | ✓ | ✓ |
| 3 |  | ✓ |  |  |  |  | ✓ | ✓ |
| 4 | ✓ |  |  |  |  |  | ✓ | ✓ |
| 5 |  |  | ✓ |  | ✓ |  | ✓ | ✓ |
| 6 | ✓ |  |  |  | ✓ |  |  | ✓ |
| 7 |  | ✓ |  |  | ✓ |  |  | ✓ |
| 8 | ✓ |  |  |  | ✓ |  | ✓ |  |
| 9 | ✓ |  |  |  | ✓ |  | ✓ |  |
| 10 | ✓ |  |  |  |  |  |  | ✓ |
| 11 | ✓ |  |  |  |  |  | ✓ | ✓ |
| 12 | ✓ |  |  |  |  | ✓ (nivolumab) |  | ✓ |
| 13 | ✓ |  |  |  |  |  |  | ✓ |
| 14 | ✓ |  |  |  |  |  | ✓ | ✓ |
| 15 | ✓ |  |  |  | ✓ | ✓ (daratumab) | ✓ | ✓ |
| 16 |  | ✓ |  |  |  |  | ✓ | ✓ |
| 17 |  | ✓ |  |  | ✓ |  |  | ✓ |
| 18 |  | ✓ |  |  |  | ✓ (rituximab) | ✓ | ✓ |
| 19 |  |  |  | ✓ |  |  | ✓ |  |
| 20 |  | ✓ |  |  | ✓ |  | ✓ |  |

Supplementary Table 2a. MRONJ outcomes and use of L-PRF across extracted teeth in polypharmacy patients

| Patient ID | MRONJ+ | | MRONJ– | |
| --- | --- | --- | --- | --- |
|  | Extracted teeth (n) | Use of L-PRF | Extracted teeth (n) | Use of L-PRF |
| 1 | 2 | ✓ | 2 | ✓ |
| 2 | 1 | ✓ | 4 | ✓ |
| 3 | 2 | ✗ | 4 | ✗ |
| 4 | 2 | ✗ | 1 | ✗ |
| 5 | 4 | ✗ | 5 | ✗ |
| 6 | 6 | ✓ | 1 | ✓ |
| 7 | 1 | ✗ | 4 | ✗ |
| 8 | 5 | ✗ | 7 | ✗ |
| 9 | 2 | ✗ | 5 | ✗ |
| 10 | 2 | ✗ | 4 | ✗ |
| 11 | 1 | ✗ | 1 | ✗ |
| 12 | 1 | ✓ | 1 | ✓ |
| 13 | 1 | ✓ | 7 | ✓ |
| 14 | 1 | ✗ | 1 | ✗ |
| 15 | 2 | ✓ | 1 | ✓ |
| 16 | 3 | ✓ | 3 | ✓ |
| 17 | 2 | ✓ | 5 | ✓ |
| 18 | 2 | ✓ | 3 | ✓ |
| 19 | 2 | ✓ | 5 | ✓ |
| 20 | 1 | ✗ | 2 | ✗ |

MRONJ+ = MRONJ development observed

MRONJ– = No MRONJ development observed

(✓) = with L-PRF

(✗) = without L-PRF

Supplementary Table 2b. Summary of MRONJ outcomes by L-PRF use in polypharmacy patients

| Use of L-PRF | MRONJ+ | MRONJ- | Total |
| --- | --- | --- | --- |
| ✓ | 22 | 32 | 54 |
| ✗ | 21 | 34 | 55 |

*Chi-square test showed no significant association.*

MRONJ+ = MRONJ development observed

MRONJ– = No MRONJ development observed

(✓) = with L-PRF

(✗) = without L-PRF
